# Differential effects of prolonged work on performance measures in self-paced speed tests.

**DOI:** 10.2478/v10053-008-0070-8

**Published:** 2010-03-03

**Authors:** Michael B. Steinborn, Hagen C. Flehmig, Karl Westhoff, Robert Langner

**Affiliations:** 1 Department of Cognitive and Biological Psychology, University of Tübingen, Germany; 2 Department of Psychology, Technische Universität Dresden, Germany; 3 Departments of Neurology and Psychiatry, RWTH Aachen University, Germany

**Keywords:** reaction time, mental fatigue, sustained performance, time on task, practice effects

## Abstract

Time-related changes in the speeded performance of complex cognitive tasks are considered to arise from the combined effects of practice and mental fatigue. Here we explored the differential contributions of practice and fatigue to performance changes in a self-paced speeded mental addition and comparison task of about 50 min duration, administered twice within one week’s time. Performance measures included average response speed, accuracy, and response speed variability. The results revealed differential effects of prolonged work on different performance indices: Practice effects, being more pronounced in the first session, were reflected in an improvement of average response speed, whereas mental fatigue, occurring in both sessions, was reflected in an increase of response speed variability. This demonstrates that effects of mental fatigue on average speed of performance may be masked by practice effects but still be detectable in the variability of performance. Therefore, besides experimental factors such as the length and complexity of tasks, indices of response speed variability should be taken into consideration when interpreting different aspects of performance in self-paced speed tests.

## INTRODUCTION

When individuals continuously perform a speedFed cognitive task over prolonged time periods, performance usually deteriorates as a function of time on task (TOT). This has been attributed to accumulating *mental fatigue*, which has been found to impair performance in a variety of cognitive tasks. In most studies on this subject, mental fatigue is used as an umbrella term that includes a decrease in arousal, motivation, and tonic activation levels, and by this means impose a deterioration of cognitive control functions (Bratzke, Rolke, Steinborn, & Ulrich, 2009; Helton & Warm, 2008; Matthews et al., 2002). In contrast, in sufficiently complex tasks, practice improves performance over time, which may compensate or even overrule performance impairments from fatigue (Hagemeister, 2007; Healy, Wohldmann, Sutton, & Bourne, 2006; Pieters, 1985). This study examined time-on-task effects on self-paced speeded performance in a continuous mental addition and comparison task by considering practice effects that are especially pronounced at the beginning and the effects of accumulating mental fatigue that may particularly affect performance towards the end of a testing session. To further disentangle the effects of practice and mental fatigue, we compared the effect of prolonged work on distinct aspects of performance, including speed, accuracy, and variability. Finally, since we are also concerned with constructing speeded tests for purposes of psychological assessment (Westhoff, Hagemeister, & Strobel, 2007), we examined the basic psychometric properties of the different facets of performance with regard to their retest-reliability and intercorrelations (Flehmig, Steinborn, Langner, Scholz, & Westhoff, 2007; Van Breukelen et al., 1996).

### Performance in prolonged self-paced speed tests

Self-paced speed tests have been employed to assess the ability to sustain mental focus and concentration over extended time periods (cf. Van Breukelen et al., 1996, for a review). Optimal performance in such tasks requires top-down control over energizing basal cognitive processes, balancing speed and accuracy, and shielding the cognitive system against task-unrelated thoughts and response tendencies (Smallwood, McSpadden, Luus, & Schooler, 2008). In contrast to so-called warned-foreperiod tasks, in which the individuals’ are enabled to establish a state of “peak” readiness at an expected moment of time but can take some rests during the intertrial-interval (Los & Schut, 2008; Steinborn, Rolke, Bratzke, & Ulrich, 2008, 2009; Wascher, Verleger, Jaśkowski, & Wauschkuhn, 1996), self-paced speed tests require the individuals to actively maintain a rather stable state of sufficient activation to accomplish the task demands (e.g., Li et al., 2004; Yasumasu, Reyes Del Paso, Takahara, & Nakashima, 2006). Because attentional top-down control is rather difficult to sustain for longer than a few seconds (Gottsdanker, 1975; Langner, Steinborn, Chatterjee, Sturm, & Willmes, in press), maintaining optimal performance levels in attention-demanding tasks over extended periods of time requires a mechanism that cyclically reactivates attentional control. This sustained optimization is considered an effortful process of self-regulation, often termed *sustained mental concentration* (e.g., Li et al., 2004; Meiran, Israeli, Levi, & Grafi, 1994, p. 729; Rabbitt, 1969; Van der Ven, Smit, & Jansen, 1989, p. 266).

Self-paced speed tests allow the assessment of different performance aspects (cf. Pieters, 1985; Van Breukelen et al., 1996). In particular, performance can be measured as average response speed, response accuracy, or response speed constancy. Depending on the particular task (e.g., its complexity, response mode, etc.), these aspects have been shown to be distinct from each other, differently predicting various criteria. For example, Flehmig et al. (2007) showed that response speed and accuracy in self-paced speed tests are largely independent dimensions of performance. Moreover, they examined the psychometric properties of response speed variability in several speeded choice tasks and demonstrated that response speed variability is a reliable measure that captures different aspects of performance than conventional measures (e.g., Pieters, 1985; Rabbitt, Osman, Moore, & Stollery, 2001; Van Breukelen et al., 1996). When individuals work continuously over prolonged time periods on a cognitive task, two opposing processes may affect their performance: On the one hand, performance might improve, becoming faster, more accurate, and less variable, as the individuals acquire the skill to optimally perform the task. On the other hand, performance might deteriorate as the individuals start suffering from the effects of mental fatigue, boredom, and reduced attention over time. Both the beneficial and detrimental effects have been documented in the literature (cf. Bratzke et al., 2009; Healy, Kole, Buck-Gengler, & Bourne, 2004; Sanders & Hoogenboom, 1970).

Fatigue effects are considered to occur because top-down control deteriorates with prolonged time-on-task, particularly resulting in more variable response speed, because involuntary rest breaks (i.e., mental blocks) during the task become more frequent whereas the fastest responses oftentimes remain stable (e.g., Archer & Bourne, 1956; Bertelson & Joffe, 1963; Bills, 1931; Bunce, Warr, & Cochrane, 1993; Sanders & Hoogenboom, 1970). According to a widely held view, these extra-long responses in self-paced speed tests arise from intertrial carryover effects that accumulate during a sequence of trials (e.g., Johnson et al., 2007; Rabbitt, 1969; Welford, 1959). That is to say, even after completing the response in the previous trial, performance is still affected by a post-response refractory period that strains processing capacity during prolonged self-paced work. Although the individuals partially compensate for this by optimizing energy expenditure, a residual bottleneck accumulates resulting in occasional interruptions of processing, as reflected by the characteristic mental blocks.

Practice effects, occurring by means of procedural learning, are considered to produce permanent changes in memory that allow the individuals to prepare serial choice decisions more quickly and carry them out more efficiently (Pashler & Baylis, 1991; Proctor, Weeks, Reeve, Dornier, & Van Zandt, 1991). Current theoretical models say that components of the task that are initially processed algorithmically (by means of controlled information processing) are then, after practice, processed in a rather automatic fashion (by means of sole memory retrieval of previously encountered stimulus-response relations). Therefore, practice effects are considered to counteract the effects of mental fatigue by masking the effects of TOT on performance (e.g., Healy et al., 2006; Logan, 1992; Pashler & Baylis, 1991). Individual differences in the susceptibility to mental fatigue or in the ability to learn from previous testing sessions or both may produce measurement artefacts that also affect the predictive validity of psychometric tests and should therefore be controlled by experimenters and practitioners (cf. Ackerman & Kanfer, 2009; Pieters, 1985; Van Breukelen et al., 1996).

### Experimental approach

The present study aimed to explore the differential effects of practice and fatigue on different measures of performance during self-paced speeded responding. In many studies on this subject, performance improved over time, indicating that the beneficial effects of practice were greater than the detrimental effects of fatigue within about 30-60 min of testing time. However, if the task was to be performed over longer time periods without rest breaks, the negative effects of mental fatigue cancelled out or even overruled the positive effect of learning. Moreover, it has been shown that practice and fatigue affect measures of performance rather differently (Healy et al., 2004). Whereas practice has been shown to have a global effect on average speed, time-related mental fatigue is considered to primarily affect response speed variability (e.g., Pieters, 1985; Van Breukelen et al., 1996, for a review).

Here we examined the changes in different performance measures with extended work in a self-paced mental addition and comparison task of 50 min task length, administered twice within a test–retest interval of one week. Notably, performance fluctuations due to extended work are especially pronounced in self-paced tasks (i.e., tasks in which an imperative signal follows immediately after the participant’s response to the previous imperative signal), since these tasks require the individual to continuously track response speed and accuracy to maintain optimal performance (e.g., Rabbitt, 1969; Rabbitt & Banerij, 1989). From this cognitive-chronometric perspective, we predicted that when rather complex tasks are used (e.g., mental addition), TOT-related practice effects should be indicated by an increase in average response speed, and this speed-up should be more pronounced at the first testing session compared to the retesting session (Compton & Logan, 1991; Healy et al., 2006). In contrast, TOT-related fatigue should especially be indicated by an increase of response speed variability (Sanders & Hoogenboom, 1970; Steinborn, Flehmig, Westhoff, & Langner, 2008).

From a psychometric perspective, response speed variability is considered as reflecting states of lowered arousal or distractibility (e.g., de Zeeuw et al., 2008; Sanders, 1998, pp. 418-426). Therefore, it has been argued that variability measures often exhibit lower test–retest reliability compared to measures of average speed and are thus to be evoked by the experimenter (Pieters, 1985; Van Breukelen et al., 1996). Following Rabbitt et al. (2001) , we further predicted that if stable (i.e., trait-like) individual differences in response speed variability exist, they should be reflected in high test–retest reliability scores. In addition, if individual differences are further increased by accumulating fatigue, this should be indicated by an increase of response speed variability as a function of TOT. Proceeding from the work of others (e.g., Flehmig et al., 2007; Segalowitz, Poulsen, & Segalowitz, 1999; Van Breukelen et al., 1996), we computed five indices of performance, namely average response speed (i.e., mean reaction time [RTM], median reaction time [RTMD]), response accuracy (i.e., error percentage [EP]), and response speed variability (i.e., reaction time standard deviation [RTSD], coefficient of variation [RTCV]). RTM and RTMD were used as an estimate of mental speed, and EP to measure the individual’s tendency to keep a certain standard of quality. RTSD and RTCV were used as estimates of distractibility (cf. Pieters, 1985; Van Breukelen et al., 1996).

## METHOD

### Participants

One-hundred and three volunteers participated in the study, which took place on two separate dates one week apart. Three participants dropped out after the first testing session and were excluded from the data set, so that 100 participants (50 male, 50 female; mean age = 26.6 years, *SD* = 7.3 years) entered the final analysis. Most participants were right-handed and all of them had normal or corrected-to-normal vision.

### Task description

**Figure 1.**
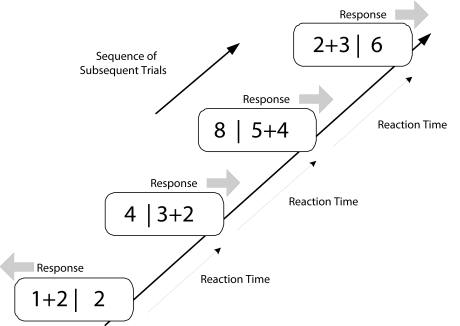
Example of a typical sequence of trials in the Serial Mental Addition and Comparison Task (SMACT). By pressing either the left or right response key, participants indicated the side of the larger numerical value. The task is self-paced, that is, the presentation of a new trial follows immediately after the previous response.

The Serial Mental Addition and Comparison Task (SMACT) was employed (Restle, 1970). This task requires participants to self-pace their responding, since each item in a trial is presented until response and replaced immediately after the response by the next item. As in other self-paced speed tests, no feedback is given, neither in case of an erroneous response, nor in case of too slow responses. In each trial, an addition term together with a single number was presented; both were spatially separated by a vertical bar (e.g., “4+5 | 10”). Participants were required to solve the addition problem and then to compare the number value of their calculated result with the number value of the separately presented digit. The value of the digit was either one point smaller or one point larger than the result of the addition but never of equal value. Participants were instructed to indicate the larger number value by pressing either the left or the right shift key as fast as possible, in accordance with the side the larger value was presented at. That is, when the value on the left side was larger (e.g., “2+3 | 4”), they had to respond with the left key, and when the number value on the right side was larger (e.g., “5 | 2+4”), they had to respond with the right key (see Figure 1).

The present version of the SMACT differed from previous ones (e.g., Steinborn, Flehmig et al., 2008) with regard to item-set size and overall testing time. In particular, we employed items with a problem size (i.e., the numerical size of the result of a particular addition problem, which directly determines the computational difficulty of the task) ranging from 4 (e.g., “2+3 | 4”) to 18 (e.g., “9+8 | 18”). A rather small set of 48 items was used. Each of the items was presented 34 times during a session, amounting to a total of 1632 randomly presented trials. For both the first and the second testing session, these 1632 trials were divided into four consecutive parts (Test Bins 1-4), so that each part contained 408 trials. These four parts were then analyzed to examine the effect of extended work on performance speed, accuracy, and variability. Altogether, the task lasted about 50 min.

### Procedure

The experiment took place in a noise-shielded room and was run on a standard IBM-compatible personal computer with color display (19”, 150 Hz frequency), using the software package Experimental Runtime System (ERTS) for stimulus presentation and response recording. The two experimental sessions took place on separate days, with a retest interval of one week. Both testing sessions were administered at normal daytimes (between 10:00 and 16:00), yet not always at the exact time of day. Participants were seated at a distance of about 60 cm in front of the computer screen, and the stimuli were presented at the center of the screen.

## RESULTS

### Data analysis

In general, correct responses shorter than 100 ms were regarded outliers and discarded from further analysis. To obtain a measure of average speed, RTM was computed as the arithmetical mean of response times. As truncation criterion, only responses shorter than 2.5 standard deviations above the individual mean were used (Ulrich & Miller, 1994). In addition, to obtain a measure of speed that is insensitive to reaction time outliers, RTMD was additionally computed as the median of response times. Incorrect responses were used to compute EP (error percentage) as an index of accuracy. The indices RTSD and RTCV were computed as measures of absolute and relative (i.e., mean-corrected) response speed variability. RTSD was computed as the individual standard deviation of response times, and RTCV was computed as RTSD divided by RTM and multiplied by 100. Since exta-long response times are particularly important to interpret variability measures (Bills, 1931; Sanders & Hoogenboom, 1970), no truncation criterion was used to compute RTSD and RTCV.

### Correlational analysis

Table 1 shows the retest reliability of all performance indices and the correlations among them. As expected, RTM and RTMD showed high retest reliability and intercorrelation. Performance accuracy (as indexed by EP) showed sufficient retest reliability and was virtually uncorrelated with performance speed (as indexed by RTM or RTMD). Likewise, mean-corrected response speed variability (as indexed by RTCV) was sufficiently reliable at the beginning (Bin 1). Interestingly, its reliability increased over time (Bin 4), indicating that the stability of individual differences was further enhanced through prolonged time on task. Notably, RTCV was somewhat intercorrelated with RTM (Flehmig et al., 2007) but virtually uncorrelated with RTMD (Table 1).

**Table 1.** Retest Reliability and Intercorrelation of Performance Measures in the Serial Mental Addition and Comparison Task (SMACT), Separately Shown for the First and Last Testing Bins

		Session 1									
		Performance at beginning (Testing Bin 1)					Performance at end (Testing Bin 4)				
		RTMD	RTM	EP	RTSD	RTCV	RTMD	RTM	EP	RTSD	RTCV
		1	2	3	4	5	6	7	8	9	10
Session 2 (Retest)	1	**.85**	**.99**	-.03	**.60**	.13	**.90**	**.87**	-.08	**.45**	.19	
	2	**.98**	**.85**	-.04	**.70**	**.25**	**.89**	**.89**	-.08	**.53**	**.29**
	3	-.11	-.09	**.68**	-.05	-.03	-.05	-.05	**.72**	.02	.04
	4	**.54**	**.68**	-.04	**.79**	**.85**	**.55**	**.65**	-.05	**.79**	**.69**
	5	**.26**	**.41**	.02	**.90**	**.73**	.11	**.23**	.01	**.65**	**.74**
	6	**.95**	**.94**	-.10	**.50**	**.21**	**.91**	**.98**	-.07	**.54**	**.23**
	7	**.92**	**.95**	-.11	**.65**	**.36**	**.97**	**.91**	-.06	**.69**	**.40**
	8	.00	.05	**.50**	.15	.18	-.02	.03	**.66**	.05	.09
	9	**.53**	**.65**	-.10	**.88**	**.75**	**.53**	**.71**	.17	**.89**	**.91**
	10	**.28**	**.41**	-.06	**.76**	**.79**	**.26**	**.45**	**.20**	**.91**	**.81**

*Note*. RTMD = median reaction time, RTM = mean reaction time, EP = error rate, RTSD = standard deviation of reaction times, RTCV = coefficient of variation of reaction times. Time bins were defined according to the amount of work, each bin containing one quarter of the whole series of trials (i.e., 408). Test–retest reliability is shown in the main diagonal; correlations for the first session are shown above, for the second session below the main diagonal. Significant correlations are denoted in bold (*N* = 100; *r* ≥ .20, *p* < .05; *r* ≥ .26, *p* < .01).

### ANOVA

**Figure 2.**
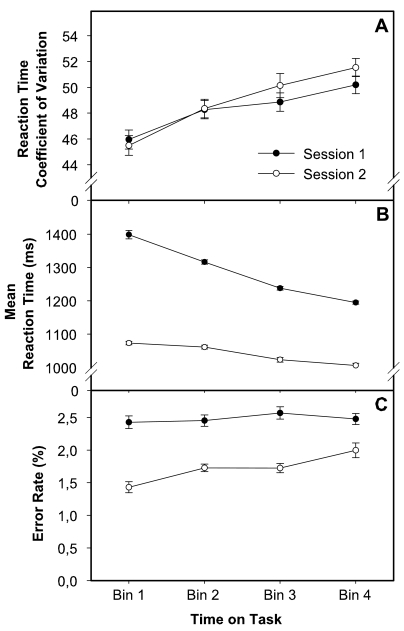
Effects of session and time on task (TOT) on performance in the Serial Mental Addition and Comparison Task (SMACT). Data are separately displayed for response speed variability (A), average response speed (B), and accuracy (C). Standard errors (error bars) are computed according to Cousineau (2005).

A two-factorial within-subject analysis of variance (ANOVA) was performed, with session (levels: test vs. retest) and TOT (levels: Bins 1-4) as factors and the respective performance indices as the dependent measures. When necessary, the Greenhouse–Geisser correction was used to compensate for violations of sphericity. Both main effects and interaction effects are listed in Table 2. Figure 2 displays RTMD, EP, and RTCV as a function of TOT.

**Table 2.** Effects of Session and Time on Task (TOT) on Different Measures of Performance in the Serial Mental Addition and Comparison Task (SMACT)

Source	*df*	*F*	*p*	η²	
Mean reaction time (RTM)				
1 Session	1,99	514,2	.000	.84
2 TOT	3,297	185,5	.000	.65
3 Session ✕ TOT	3,297	86,0	.000	.47
Median reaction time (RTMD)				
1 Session	1,99	550,3	.000	.85
2 TOT	3,297	205,1	.000	.67
3 Session ✕ TOT	3,297	79,6	.000	.45
Error percentage (EP)				
1 Session	1,99	73,6	.000	.43
2 TOT	3,297	3,8	.023	.04
3 Session ✕ TOT	3,297	3,6	.015	.04
RT standard deviation (RTSD)				
1 Session	1,99	44,8	.000	.31
2 TOT	3,297	0,73	.533	.00
3 Session ✕ TOT	3,297	5,7	.002	.05
RT coefficient of variation (RTCV)				
1 Session	1,99	0,3	.569	.00
2 TOT	3,297	18,1	.000	.15
3 Session ✕ TOT	3,297	1.2	.304	.1

*Note*. Effect size: partial η²; TOT = time on task (Time Bin 1-4); Session (test vs. retest).

As predicted, the factor TOT had a significant effect on performance: Reaction time decreased within a session, indicating that learning occurred during the test, *F*(3, 297) = 185.5, partial η² = .65, *p* < .01. The session effect on RTM revealed a significant intersession improvement, *F*(1, 99) = 514.2, partial η² = .84, *p* < .01. The session × TOT interaction effect on RTM, *F*(3, 297) = 86.0, partial η² = .47, *p* < .01, indicated that learning during the test was larger at the first testing session (unpractised condition: RTM_1_ to RTM_4_ = 1399, 1316, 1238, 1195 ms) than at the second testing session (1073, 1061, 1023, 1006 ms). The ANOVA results for RTMD as dependent measure were virtually the same. With respect to the error rate (EP), TOT had an entirely different effect, since the percentage of errors increased over time, *F*(3, 297) = 3.8, partial η² = .04, *p* < .05. The effect of session on EP, *F*(1, 99) = 73.6, partial η² = .43, *p* < .01, showed that response errors occurred less frequently at retest (i.e., after practice) compared to the first testing session. The TOT effect on EP was qualified by a crossed session × TOT interaction, *F*(3, 297) = 3.6, partial η² = .04, *p* < .05, which indicated that the number of errors actually remained stable during the first session (EP_1_ to EP_4_ = 2.4%, 2.4%, 2.5%, 2.4%), and increased only after practice, that is, during the second testing session (1.4%, 1.7%, 1.7%, 1.9%).

Further, mean-corrected response speed variability (RTCV) also increased during the task, *F*(3, 297) = 18.1, partial η² = .15, *p* < .01, indicating that very slow responses occurred more frequently toward the end of a testing session (Session 1: 46.1%, 48.4%, 49.0%, 50.3%; Session 2: 45.6%, 48.4%, 50.2%, 51.6%). Notably, this occurred even though average response speed became faster, demonstrating a dissociation between measures of average response speed and response speed variability. There was no main effect of session and no session × TOT interaction effect on RTCV, indicating that this measure is less sensitive to practice than indices of average response speed. It should be noted that the results did not change when we defined the four testing bins per session according to the exact individual time at work instead of defining it according to the amount of work (i.e., the number of trials).

Taken together, the ANOVA results demonstrated a divergence between measures of speed and measures of accuracy and variability over 50 min of prolonged self-paced speeded performance (Li et al., 2004; Yasumasu et al., 2006). Interestingly, the decrease in average reaction time (RTM) as well as the increase in variability (RTCV) appeared to occur quite monotonously during TOT. Accordingly, post-hoc (single contrast) comparisons revealed that differences were largest between time Bin 1 and 4 for both RTM, *F*(1, 99) = 236.8, partial η² = .71, *p* < .001, and RTCV, *F*(1, 99) = 34.0, partial η² = .26, *p* < .001. Further, RTCV appeared to be robust against between-session and within-session practice effects, which might have masked potential effects of mental fatigue on measures of average performance speed (Figure 2).

## DISCUSSION

Our study investigated how mental fatigue from prolonged work affects performance in self-paced speed tests. To this end, we examined the effect of time on task (TOT) on the speed, accuracy, and variability of responding in a 50-min version of the SMACT. The results revealed differential effects of TOT on different performance indices: Practice effects chiefly occurred in the first session and were reflected in an increase of average response speed (i.e., RTM and RTMD), whereas mental fatigue effects, which can be assumed to occur in both sessions, were reflected in an increase of response speed variability (i.e., RTCV). As predicted, practice-related increases in average response speed were larger at the first testing session. In contrast, fatigue-related increases in error rate (i.e., EP) were present only at the second testing session. The fatigue-related increase in response speed variability (RTCV) was about similar at both testing sessions.

The present study corroborated the utility of RTCV as an “attentional-state index”, as suggested previously (e.g., de Zeeuw et al., 2008; Segalowitz et al., 1999)^1^. RTCV appeared to be selectively sensitive to the detrimental, fatigue-related effects of prolonged responding – in contrast to measures of average speed, a strong increase over time was found, indicating growing distractibility (Pieters & Van der Ven, 1982; Smit & Van der Ven, 1995). This sensitivity to mental fatigue is confirmed by its retest reliability which increased with TOT (from *r* = .72 to *r* = .82). This increase indicates that the most stable individual differences were evoked towards the end of the prolonged continuous work, when the detrimental effects of accumulating fatigue presumably affects performance most (Helton & Warm, 2008; Smulders & Meijer, 2008). Although the effect of TOT on performance variability was rather small, the present study is the first to directly show a dissociation, or divergence in the direction, between measures of speed and variability due to changes in the individuals’ attentional state.

The significant increase of RT variability with TOT does not only replicate previous results on mental blocks (Bunce et al., 1993; Sanders & Hoogenboom, 1970), but extends this research by showing that accumulating short-term fatigue is reliably captured by psychometric measures of response speed variability (i.e., RTCV). Thus, the results provide evidence for the impact of mental fatigue on performance efficiency in self-paced cognitive tasks. Previous research supports the notion that instability of cognitive control functions is a major cause for this deterioration of performance stability, although a decrease in arousal and intrinsic motivation may also play a role, especially in highly repetitive situations like the present one. Here we did not intend to dissociate the different facets of mental fatigue but aimed to examine the differential effect of TOT on different performance measures, including changes in their psychometric properties. However, further research is needed to disentangle separate effects of these and other energetic variables (e.g., diurnal and circadian rhythms) and to examine the effects of stronger modulations, for example, under conditions of sleep deprivation or during shift-work schedules (Bratzke et al., 2009).

The percentage of errors was stable at the first testing session but increased during TOT at retest. At first glance, this seems surprising, since improvements due to practice should protect the individuals from making too many response errors. We suggest that lowered motor responsiveness yielded this paradoxical result, such that impulsive reactions become especially pronounced with higher degrees of automaticity during a task (i.e., because responses are then based on stimulus-response associations, Compton & Logan, 1991; Healy et al., 2006). Under normal conditions, this typically results in faster responding. Under fatigued conditions, however, an increase in error rate can also be expected (Healy et al., 2004). It should be noted, however, that overall error rate was especially low in the present study, which is typically observed in self-paced tasks (Rabbitt, 1969). For example, when the response–stimulus interval is much larger (e.g., up to 600 ms), a higher overall error rate would be expected, and TOT could probably have a more pronounced effect on error rate (and a smaller effect on response speed variability).

The use of rather complex stimulus material may have contributed to the result pattern obtained for RTM, since practice effects counteracted the time-related performance decline that is typically observed in simple and highly compatible or overlearned choice reaction-time tasks. This conclusion is supported by earlier studies using stimuli differing in complexity. For example, Compton and Logan (1991) showed that learning benefits were stronger and occurred more quickly for difficult items than for easy ones and for small item sets than for large ones, respectively. In research on energetic variables such as field studies on shift work (Bratzke et al., 2009), or in applied testing situations such as in the context of personnel selection (Hagemeister, 2007), practice effects may mask the effects of the variables under scrutiny and thus have to be strictly controlled by the experimenter (Flehmig et al., 2007; Healy et al., 2004).

Alternatively, measures should be selected that are less sensitive to practice but still reflect the impact of energetic changes. Our results clearly show that only average response speed improved during continuous mental work but not accuracy and response speed variability. This is consistent with the view that accumulating mental fatigue is better reflected in measures of performance variability rather than average performance speed (de Zeeuw et al., 2008; Hayashi, 2000; Stuss, Murphy, Binns, & Alexander, 2003). It should be noted that previous studies on self-paced work were mainly concerned with the frequency of mental blocks (Bertelson & Joffe, 1963; Bunce et al., 1993), which are suitable to measure experimental effects but are problematic in psychometric testing. For example, Bills (1931) defined *mental blocks* as responses longer than twice the mean, others as responses longer than twice the median (e.g., Bertelson & Joffe, 1963; Weaver, 1942) However, frequency measures of blockings have been shown to lack reliability, most probably because they are built on only a small proportion of responses relative to the entire RT distribution (Van Breukelen et al., 1996). Therefore, a major contribution of the present study is the measurement of TOT-related performance fluctuations by means of psychometrically suitable variability measures, assessing not only the experimental effects of TOT but also their applicability in psychometric testing.

## Conclusions

Using an extended version of the SMACT, which required self-paced speeded performance over a period of about 50 min, we showed a dissociation between practice and fatigue effects on different performance measures. Precisely, whereas RTM and RTMD decreased over the testing session due to practice, RTCV increased due to mental fatigue. This suggests RTCV as a useful index for detecting fatigue in applied testing situations, particularly in personnel selection and school psychology. Since performance in different speed tests typically is highly intercorrelated (Flehmig et al., 2007), the present results can be generalized to other forms of self-paced choice reaction tasks of about the same complexity. By means of sensitive measures that can be derived from any such task, suboptimal states of mental functioning may potentially be detected and taken into account, improving the predictive validity of performance measurements, both in basic research and in applied testing situations.

## Footnotes

1 Concerning the attentional-state index: It has first been argued by Bills (1931) and later by Sanders (1998, pp. 418-426) that RT variability is a “state measure,” particularly reflecting states of lowered arousal. This can be caused by situational factors such as sleep deprivation (Bratzke et al., 2009) or pharmacological effects (Hayashi, 2000), but can also result from an inherent trait characteristic. For example, this view has been supported by studies on attention-deficit/hyperactivity disorder (ADHD): Children with ADHD are sometimes variable in their responding, sometimes not, depending on their particular attentional state at the moment of testing. That is to say, these individuals are more frequently distracted than healthy participants, but not necessarily at any given testing sessions (cf. de Zeeuw et al., 2008; Johnson et al., 2007; Sanders, 1998, pp. 418-426). The same is true for individuals with high neuroticism levels, but here variability is evoked by worries and state anxiety, which are not observed every day to the same extent (Robinson, Wilkowski, & Meier, 2006). We here tested whether a state of lowered arousal/stronger fatigue can be experimentally induced in normal individuals, and whether this would be reflected in higher RT variability.
